# Embitterment – Conception of a Potential Moderator to Dysfunctional and Aggressive Behaviour in Children and Adolescents

**DOI:** 10.1007/s40653-021-00407-4

**Published:** 2021-10-11

**Authors:** Tim Balder, Michael Linden

**Affiliations:** Dept. of Psychosomatic Medicine, Charité University Medicine Berlin, CBF, Hs IIIA, Hindenburgdamm 30Rm 13/14, 12200 Berlin, Germany

**Keywords:** Embitterment, Justice, Humiliation, Children and adolescents, Breach of trust

## Abstract

Embitterment is an emotion which is known to everybody in reaction to injustice, humiliation, and breach of trust. Children and adolescents have an understanding of justice/injustice and fairness, violations of injustice, humiliation, and breach of trust are also stressors at a young age. In this conceptual paper it is argued that embitterment is also seen in children and adolescents, and that parents, educators and therapists should recognize this emotion. This could possibly help to early identify children at risk for severe dysfunctional and aggressive behaviours, when preventive interventions are still possible. The article concludes with concepts on how to diagnose and treat children with embitterment.

## Phenomenology of Embitterment

Embitterment is a universal human emotion, which is known to everybody, similar to anxiety, love, sadness, or anger (Linden & Maercker, 2011). About every second person in the general population reports feelings of embitterment, when they were asked to think about negative life events in the past (Baumann & Linden, 2015). Embitterment occurs in reaction to injustice, humiliation, and breach of trust, which are predominant social stressors in adulthood (Bülau et al., 2016). The psychoanalyst Alexander (1960) describes embitterment as a self-reinforcing “masochistic adjustment reaction” that gives a sense of control, even if this means self-destruction. Injustice can be seen as a form of aggression, while embitterment is a kind of counter aggression with the desire to reinstall justice and defend one’s self worth. It is a burning emotion combined of feelings of frustration, self-reproach, down heartedness and wishes for revenge (Znoj et al., 2016). It can occur as normal emotion, as part of some other mental illness, and in greater intensity, it can constitute a mental disorder in the form of “post traumatic embitterment disorder (PTED)” (Kühn et al., 2018; Linden, 2017; Linden & Arnold, 2021; Linden & Noack, 2018; Linden & Rotter, 2018).

It has been shown that the clinical recognition of embitterment in adults is important, as this emotion is frequent and can explain psychological suffering, dysfunctional and sometimes even dangerously aggressive behaviour. In general, it can severely affect a person's life, can cause sickness leave as well as workplace problems, and is in need of special treatment (Kühn et al., 2018; Michailidis & Cropley, 2017; Muschalla, 2021; Sensky et al., 2015; Valdes-Stauber et al., 2015). Conner and Weisman (2011) postulate that people, who are more susceptible for embitterment have a higher risk for suicide.

There is to our knowledge no research in this regard in children and adolescents. This is surprising, as the experience of injustice, humiliation and bullying can be regularly observed at a younger age (e.g. Coie & Dodge, 1983; Correia & Dalbert, 2007; OECD, 2017), which indicates that there could also be embitterment. This topic has been neglected so far by both clinical and developmental researchers, although it has the potential to better understand children's and adolescents' maladaptive reactions to social stressors. It is of particular relevance, as embitterment-like reactions are potentially highly maladaptive and thus deserving of closer scrutiny.

In this paper, we want to review the potential relevance of embitterment in children and adolescents in order to stimulate theory-building and future research.

## Injustice, Humiliation, and Breach of Trust in Childhood

There is ample evidence that feelings of injustice, humiliation, and breach of trust are severe and frequent social stressors in young age. Childhood does not only consist of happiness. Children have scary, depressing, frustrating, or even frightening experiences. Some of these are part of a normal development, such as the fear of school exams or rivalries and fights between siblings. Others are extraordinary and painful, such as being bullied at school or being a victim of domestic violence. Some experiences may even be traumatizing. In the Pisa study (OECD, 2017), it was found that on average 16 percent of 15-year-old children in Germany were at least several times per month victims of some sort of bullying. Additionally, the German crime statistic (BIBH, 2019) reported that children and minors were victims of a crime in 154.765 cases in the year 2018.

The desire for justice and severe emotional reactions to injustice can already be observed in small children. Children have, beginning with the age of just 15 months, a sense of inequality and fairness (Geraci & Surian, 2011). A judgement on merit and justice already develops at the age of two to three years (Baumard et al., 2012; Ng et al., 2011; Sloane et al., 2012). Children want, from an early age on, that everyone gets what they deserve. According to Piaget (1997), children up to the age of seven to eight, believe in immanent justice. They expect that one will be punished in the immediate aftermath of a negative action. Over time children learn that injustices exist in the world and that innocent people can be punished or that sometimes the guilty will not be penalized. According to Dalbert and Radant (2004), a belief in a just world develops from the age of seven. The "Belief in a Just World" (Lerner, 1980) is an inborn cognitive schema, which is the basis of most social interactions. A distinction can be made between the belief that oneself should be treated with justice, which is called the personal belief in justice, and a general belief in a just society or world (Dalbert, 1999). Children are able to integrate random or unfair punishments into their concept of justice without losing faith in a just world. The belief in a just world helps individuals to have a positive view and find meaning in their lives, trust in the world and allow positive expectations for the future (Dalbert, 2001, 2009). Familial, extra-familial, and school experiences can shape what is seen as unjust (Dalbert, 2013). Liebig and May (2009) propose four models following different justice principles for social relations, which children tend to use in different social situations. For one, children learn that there are hierarchies and the "principle of rights", saying that individuals with a higher position are entitled to a larger share of goods. However, they learn an "equivalence principle", which assumes that everyone has the same rights. Additionally, there is the "performance principle" or an “economic exchange” saying that you get as much as you are willing to give yourself. Finally, there is the “achievement principle” where everyone tries to achieve the maximum profit for themselves (Liebig & May, 2009). In the third World Vision Study, Andresen et al. (2017) surveyed 2,535 children between the age of 6 and 11 years and questioned them about those four principles. The authors found that two-thirds of the children had a clear sense of injustice and that older children, girls, and those of lower social status were more sensitive to injustice.

Acts of injustice are regularly associated with humiliation, loss of face, disgrace, embarrassment, or breach of trust. As for example, similar to being able to perceive justice and injustice, children know the feeling of being humiliated far too well, as for instance humiliation is often used by bullies to obtain power and degrade others (Evans & Smokowski, 2016). As a result, children who have the feeling of being humiliated or rejected tend to act out dysfunctional and aggressive towards others (Cucu Ciuhan, 2019). In a large study in the United Kingdom, the authors found that about one fifth of children and young adults have had experienced humiliation or psychological attacks in the past (Cawson et al., 2000). Coie and Dodge (1983) even report that about one in two children have had experiences of social exclusion and bullying.

In summary, it is well established that children can distinguish different aspects of justice and injustice from a young age on and hold a belief in a just world. They show strong emotional and behavioural reactions to injustice, breach of trust, and humiliation. The hypothesis therefore is that embitterment like reactions should also be present in minors.

## Embitterment Like Reactions in Childhood

From a casuistic perspective, parents and educators know that children can show severe emotional outbursts, when they believe they are being treated unfairly. This can even go to the point that they ponder the idea to freeze to death in the snow in order to punish their unjust and overwhelming mother. This is exactly how Alexander (1960) sees embitterment as aggression by accepting self-destruction. Older children may even run amok, because of injustice and humiliation, disregarding all consequences, even at the risk of losing their own life. Minors can also be perpetrators and can even become criminals (BIBH, 2019).

Correia and Dalbert (2007) report, that students get distressed as a reaction to experiencing unjust behaviour by their teachers or other students. Bitter-like responses can also be observed in sibling rivalry, when children are in competition with each other and demand justice and a fair distribution of goods and affections. If the expectations of justice are violated, reactions like aggression, jealousy, competitive behaviour, or hatred may occur. Caffaro and Conn-Caffaro (2005) and Caffaro (2013) noted that if one sibling perceives or believes that parental preferential treatment of the other is not justified, abusive tendencies towards the other sibling together with emotional outbursts are highly likely. Effects of this abuse can be amongst other things a higher level of anxiety and depressed mood (Graham-Bermann et al., 1994; Stocker et al., 2002; Wiehe, 1990). In general, high rates of sibling violence can be an alert to psychopathological problems in the future (Lopes et al., 2019).

Children and young adults not only perceive injustice, humiliation, and breach of trust in a similar way as adults, but also tend to punish immoral and unfair behaviour even more strictly than adults (Gummerum et al., 2009). In primary school, children are mostly motivated by inequality aversion and prefer fairness and an equal distribution of goods (e.g., Blake & McAuliffe, 2011; Castelli et al., 2014; Hamann et al., 2011; LoBue et al., 2011; Warneken et al., 2011). This has been shown in experiments with the ultimatum game, where one child can propose a division of a fixed number of resources, for example candy, but the recipient (the other child) can reject the offer. As a result, neither player receives any amount of candy. Therefore, when the offer is unequal, recipients face a forced choice: one option would be to accept the inequity or to reject it, resulting in an equitable outcome of zero for each player. Studies have shown that children reject unequal offers in the ultimatum game more often than older adolescents or students (Sutter, 2007), even if this means to give up advantages for themselves. As a result of perceived injustice children and young adolescents did not only express direct punishment, but also third-party punishment, punishing unjust behaviour where one is only an unaffected observer to the situation (Kenward & Östh, 2015; Riedl et al., 2015; Gummerum et al., 2019). In this regard, older children and adolescents are more willing to punish unjust and unfair allocations than adults (Gummerum et al., 2009). Thus, inequity aversion plays a major role in why adolescents and children punish other individuals for their unjust behaviour.

Nucci and Turiel (1978) studied the response of preschoolers to the observation of moral violations and observed that children responded to rule violations first by trying to enforce correct behaviour. Additionally, they also showed violent and dysfunctional emotional reactions and withdrawal. In school children, Donat et al. (2014) observed an increased rate of immoral behaviour, expressed through cheating in examinations, if they felt, they were treated unfairly in school. Similar reactions can be observed in the course of humiliation and injustice under the keyword bullying (Stecher et al., 2018). The reaction of a child to those situations is often to first try to engage in social groups, join clubs, work with others, and in general perceive others more positively (Maner et al., 2007). If this is not successful, typical reactions are depression, anxiety, psychosomatic symptoms, helplessness, dysfunctional behaviour, or suicidal actions (Gini & Pozzoli, 2009; Haid-Stecher et al., 2018; Moore et al., 2017). Typical results of long-term social exclusion are feelings of loneliness, depression, aggression, and even suicide (Fox et al., 2015; Schoeler et al., 2018). Similarities can be seen in the Rejection Sensitivity Model (Downey & Feldman, 1996) which states that an individual who is rejected multiple times may develop expectations of rejection, consequently feeling rejected more easily and resulting in negative emotional responses such as anger or depression (Beeson et al., 2020). Studies have also shown the severe consequences, emotional abuse has on children and young adults (Dye, 2019). The parallels to embitterment are striking.

In puberty, the problem becomes even more severe. Leary and colleagues (Leary et al., 2003, 2006) investigated different school shootings (Table 1) and found that rejection, exclusion, and harassment were described as precursors in many cases. Hoffmann and Allwinn (2015) found that in two-thirds of the examined cases, the perpetrators saw the shootings as a kind of last resort, similar to how embittered adults see their revenge as a last resort to restore justice without considering the consequences (Linden, 2017). This is also comparable to the model postulated by Znoj (2011), who describes embitterment as an emotion which develops when one is hopeless and has limited external locus of control.

**Table 1:**
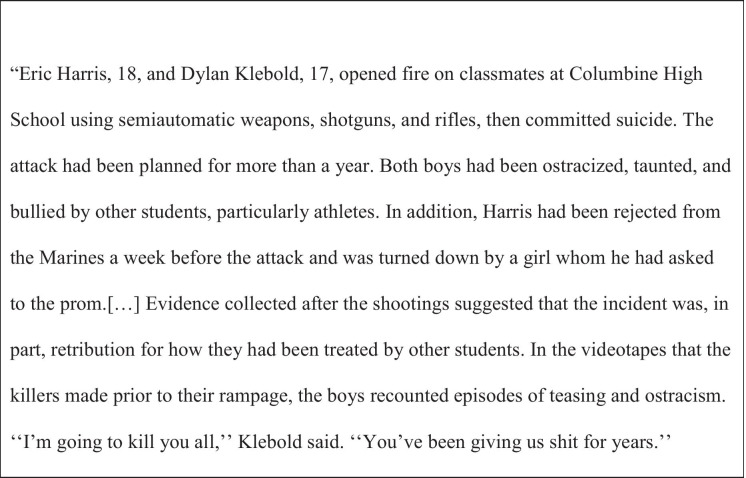
Report on the Columbine massacre by Leary et al. (2006, p. 207)

In summary, it can be said that experiences of injustice, humiliation, or breach of trust during childhood result in similar negative psychological consequences as in adulthood. Embitterment can also be observed in childhood. The problem is undoubtedly there.

How are such reactions to injustice, humiliation, and breach of trust classified in the International classification of Mental Disorders (ICD-10) (WHO, 2001)? Categories which seem to be related are the “conduct disorders (ICD-10, F91)” and the “oppositional defiant and emotional disorder (ICD-10, F91.3)”. When justice and/or goods are expected and cannot be achieved, because another person denies or obstructs this, feelings of helplessness and disappointment arise, which can lead to violent defiant reactions in children (Eisenberg & Fabes, 1992). These include strong feelings of anger, rage and stubbornness. Children tend to resist or hide and escape the situation. As children grow older, defiant behaviour manifests as oppositional behaviour. In low intensity, this is a normal expression of self-consciousness and assertiveness and is part of the early child temperament (Keenan & Shaw, 2003; Loeber et al., 2009). In greater intensity it becomes an oppositional defiant disorder. Characteristic features are recurrent negative, defiant, disobedient and hostile behaviours, especially towards peers and authorities (e.g., Rowe et al., 2010; Stringaris et al., 2010). Again, there is angriness, rage, stubbornness, resistance, unresponsiveness, and dysfunctional acts disregarding all negative consequences and dangers. This is remarkably similar to embitterment in adults (Linden, 2017).

Embitterment can last for some time. For example, running amok is not an impulsive act, but is typically planned with a long forerun (Leary et al., 2006). The early identification of embitterment could therefore open possibilities to intervene at an early stage. But as embitterment is a hybrid emotion, which encompasses general aggression towards the world and phantasies of revenge, cynicism, social retreat, and even ideas of suicide and extended suicide, this includes to repel helping hands (e.g., Linden, 2017; Linden & Noack, 2018). The question therefore is, how to help such children and adolescents.

## Coping with Embitterment in Children

The present state in clinical practice and research is, that embitterment has found no recognition in child psychology and psychiatry so far. But it might be of importance to look for this emotion, as it can already be observed in young persons and could have a predictive value for dysfunctional behaviour. This could also break new ground for the development of new treatments.

The first diagnostic step is to be sensible for general negative emotional reactions, ranging from a drop in performance at school, somatic complaints, changes in affect, social withdrawal, up to open aggression towards others or themselves. In such cases it might be recommended to clarify the context especially regarding experienced injustice, humiliation, and breach of trust. Afterwards the emotional spectrum should be explored in greater detail, with the focus on possible embitterment.

As embitterment is a nagging and destructive emotion with a tendency to persist and increase the longer it lasts, special therapeutic interventions could be indicated. Treatment of embitterment is often a challenge as patients are acrimonious towards the world and often reject help (e.g., “The world will see what it did to me”) (Linden, 2017).

Currently, there are, to our knowledge, no studies on how to treat young persons with embitterment. Hence it might be useful to connect with what is known in the treatment of embittered adults. A special approach is to refer to wisdom psychology (Linden et al., 2011). Wisdom can be described as an expertise in dealing with difficult life issues and a resilience factor in dealing with negative life events (Staudinger & Baltes, 1996). Like assertiveness, it is a multidimensional psychological capacity (Linden, 2014, 2017), including a special view to the world (factual knowledge, contextualism, value relativism), of other people (change of perspective, empathy), of one’s own person (relativization of problems and demands, self-relativization, self-distance), of one’s own experience (perception and acceptance of emotions, serenity), and the future (forgiveness and acceptance of the past, uncertainty tolerance, sustainability).

These sub-dimensions can help to overcome embitterment. In the context of child psychotherapy, the therapist could first help the child to describe in detail what happened (“factual knowledge”) and clarify the exact circumstances (“contextualism”). Important, but difficult, could be “value relativism”, which states that there can be quite different evaluations of the same situation, depending on different values of the individual. For example, if a child gets mediocre grades. On the one hand getting bad grades can be very devastating for a child, as it could have performed far better. But on the other hand, a child can also value the situation positively, because it could have done worse as well. The result could be a more complex but realistic understanding of the processes, because emotions and wishes often prevent a realistic perception of the processes.

The next step in the therapy of embittered children or adolescents would be to promote a change of perspective and to improve empathy (Baumann & Linden, 2008). This does not mean accepting what has been done, but understanding what has happened. It can be seen as an attempt to explain the motive of the perpetrator, what made him do what he or she did. This includes understanding the other person's emotions (Linden et al., 2004). The victim may wish that the perpetrator feels guilty, but in reality, he may feel proud instead. Children and adolescents are able to understand other people (e.g. Allemand et al., 2015; Doherty, 2008; McLaren et al., 2019). This does not mean that the other person is right, but it is helpful to understand what drives the other person. It is necessary to understand the perpetrator, if the victim wants to defend himself.

It may also be advisable to clarify one’s own position. What is it that I strive for, why do I think I deserve it or not? What does it really mean if I do not get it (“relativization of problems and demands”)? What are my rights when I look at it from the outside perspective (“self-relativization”)? What do other people say about my behaviour (“self-distance”)? Children understand that they cannot always be the center of the world (Kesselring & Müller, 2011). They can put their own needs into perspective and, if necessary, put them behind the needs of others (Doherty, 2008). Children also understand that not everything can happen according to their own will. This can be developed in a supportive way.

Since insults, injustice, and exclusion can lead to burning emotions, as shown above, it is essential to consider the whole spectrum of associated feelings. There might be shame, anger, the desire to destroy, and other socially unacceptable feelings (Linden, 2017). It can be helpful to name and accept them all (“perception and acceptance of emotions”) (Linden et al., 2011). Even small children can control themselves and their temperament to some extent (Zeman et al., 2006), so that they also have some influence on such reactive emotions, if they are given the opportunity to talk about them freely.

Finally, a look into the future could be helpful. How long will I stay in contact with this teacher? If I am successful later on, will it still have an impact on me? Is it useful to look back, since nobody can change what has happened (“acceptance of the past”)? What can I gain if I look ahead instead (“long term perspective”)? Forgiveness does not mean to justify or reconcile, it is a very personal decision to close the past and letting go of revenge (Freedman & Zarifkar, 2016). Children tend to be more present oriented and only in adolescence tend to become more future-oriented (Nurmi, 1991; Steinberg et al., 2009), so that they may find it easier than adults to leave things behind. The future can be used to heal the present. Additionally, one may consider aspects of hope theory (Snyder, 2002) such as the improvement of pathway thinking or agency thinking as valuable assets in the therapy of possible embittered children or adolescents.

Wisdom is not a question of age, but can be found also in children and young persons (Glück et al., 2012). Wisdom therapy, guided by the outlined wisdom dimensions, could help to process what has happened and to overcome embitterment.

## Conclusions

This is a theoretical review and conceptual paper. The goal is to stimulate a scientific and clinical discussion on embitterment, which is underrecognized and undertreated in children and adolescents. There is a need for further research in many realms. Measurements for embitterment which can be applied at a young age are missing. Epidemiological studies are needed. Conduct disorder and oppositional defiant and emotional disorders should be studied in the light of embitterment. Problems like rivalries, bullying, disgrace, downgrading, insults, humiliation, and injustice should be analyzed regarding their capacity to induce embitterment. The sensitivity of parents and educators for embitterment could possibly help to identify children at risk for severe dysfunctional and aggressive behaviours at an early stage when preventive interventions are still possible. More research in this area could also help to develop respective pedagogic and therapeutic strategies. In summary, we think that embitterment is a promising topic for future research in the field of child and adolescent psychiatry and psychology.

## Data Availability

Not applicable.
